# AI in Bundesliga match analysis—expected possession value (EPV) vs. expected goals (xG) to predict match outcomes in soccer

**DOI:** 10.3389/fspor.2025.1713852

**Published:** 2025-11-10

**Authors:** Leander Forcher, Leon Forcher, Alexander Woll, Stefan Altmann

**Affiliations:** 1 Institute of Sports and Sports Science, Karlsruhe Institute of Technology, Karlsruhe, Germany; 2 Match Analysis Department, TSG 1899 Hoffenheim, Zuzenhausen, Germany; 3 TSG ResearchLab GGmbH, Zuzenhausen, Germany

**Keywords:** football, team sports, match prediction, performance analysis, tracking data, machine learning

## Abstract

With an increasing number of key performance indicators (KPIs) in soccer analytics, it is key to identify the most valuable KPIs. One approach to define a KPI's value is to assess its ability to predict match outcomes and future performance. Therefore, this study aims to compare the effectiveness of expected goals (xG) and expected possession value (EPV) in predicting match outcomes in both pre-match and post-match scenarios. Event and tracking data of three Bundesliga seasons (2022/23, 2023/24, & 2024/25) were used to develop four distinct match outcome prediction approaches: xG & EPV pre-match (using features including the last three match performances of teams & contextual factors) and xG & EPV post-match (using xG and EPV performances of the played match). The xG post-match prediction showed the best performance in predicting match outcomes (xG post-match: RPS = 0.148, Accuracy = 0.656; EPV post-match: RPS = 0.191, Accuracy = 0.596). In pre-match scenarios EPV showed higher prediction performance (RPS = 0.194, Accuracy = 0.583) compared to xG (RPS = 0.199, Accuracy = 0.556). Accordingly, xG holds more valuable performance information on the offensive performance of a team in post-match scenarios. In contrast, the EPV pre-match prediction showed powerful results in predicting future match outcomes and thereby showcased the predictiveness of EPV.

## 1 Introduction

It is still chance that dominates the results in the game of soccer (1). This includes seemingly weaker teams regularly taking their unlikely chance by overcoming stronger opponents in knockout cup competitions. Recently, this was true for Arminia Bielefeld, playing in German third division, advancing to the final of the 2025 German DFB-Pokal defeating four first division teams including 2023/24 double winners Bayer Leverkusen. Those unlikely journeys are one of the reasons why soccer is one of the most popular sports in the world (2).

Although uncertainty is omnipresent, the coaching staff and match analysts work relentlessly to tip the probabilities in their team's favor. In this context and the latest advancements in data collection, processing, and evaluation in elite soccer, data science has become a promising field for analyzing soccer performances to find that pivotal pinpoint that flips the odds (3).

With it, a flood of key performance indicators (KPIs) have been developed and introduced to potentially bring up hidden information to gain a competitive edge. One of the most widely recognized and commonly used KPIs in soccer analytics is expected goals (xG) (4–6). While a well calibrated xG model can effectively assist in analyzing the game in more detail, it focuses exclusively on shooting actions and thereby covers only a small subpart of the complex game of soccer. This is one of the reasons why further approaches in soccer analytics shifted the focus on the analysis of every action on the pitch, for instance by modeling the scoring probabilities of possessions, namely by expected possession value (EPV). While both EPV and xG approaches (EPV & xG) primarily focus on offense, there have also been efforts to quantify defensive success (7). For instance, by analyzing the probabilities to regain the ball in defense, namely expected ball gain (xBG) (8, 9).

Next to those examples of KPIs to analyze the match performance in soccer, countless metrics are introduced and analyzed. However, the volume of these statistics can become overwhelming when trying to analyze the game through numbers and KPIs. Therefore, in the current stages of data and match analysis it is crucial to identify which KPIs contain the most valuable information (10). One effective approach to identify the most valuable KPIs is by assessing their ability to predict outcomes and future performance, making their predictive power a central aspect of their effectiveness in sports analysis (10).

While it is possible to predict several different performance aspects in soccer [e.g., running distance (11)], the prediction of match outcomes remains a central focus when forecasting future performances (12, 13). Thereby, the prediction of match outcomes can be approached from two distinct perspectives.

On one hand, from a betting market view-point it is the main aim to predict the outcomes as accurately as possible in order to maximize profit (14). Most scientific studies in this research area focus on finding insufficiencies of the betting market (15, 16). On the other hand, and in contrast to the viewpoint of the betting market, match analysis focuses on predicting outcomes to judge a player's or team's performance (e.g., by comparing pre-game predictions with actual player or team performances). With it, coaching staff is able to more objectively evaluate performance by filtering elements of chance. In this context, O'Donoghue et al. (17) showed that data analysis is more accurate in the prediction of tournament outcome than experts opinion which underlines the importance of objectively judging performance.

While betting market predictions and match analysis predictions differ in their perspectives and aims, there are also two distinct approaches to forecast outcomes. In detail, the prediction approaches of match outcomes can generally be differentiated into pre-match predictions (which include solely information available prior the match is played), and live or post-match predictions (which also include information of the performance during the played match).

In detail, pre-match prediction approaches have used highly heterogenous sets of input data dependent on the competition and available data (e.g., different data providers) (13). Thereby, the features used to predict outcomes range from rather basic information, such as league ranking of opponents or match venue, to complex input information, such as tracking data and complex tactical KPIs (18). Furthermore, there are differences in the outcome of the predictions. For instance, direct predictions of probabilities result in cumulative probabilities of the match outcome (probabilities of home win, draw, away win) (18). Indirect predictions first estimate the number of goals scored by each team, then derive match outcomes by estimating the most likely result (15, 19). This approach results in more detailed insights by modeling underlying score distributions (e.g., exact predicted goal difference).

In contrast to pre-game predictions, post-match prediction approaches use information of the match performance of teams during the actual match with the main aim to judge the performance of individual players and teams during or after a match (16). In this context, many studies in this research area have been conducted from a sports-science perspective. Thereby, a substantial set of features related to performance was used to predict match outcomes to identify which KPIs are related to match outcome performance (e.g., number of shots or possession ratio) (20, 21).

Overall, to the best of the authors’ knowledge, there has not been a study that specifically analyzed and compared the prediction performance of KPIs, namely xG and EPV, to eventually evaluate their usefulness to analyze the game of soccer.

Therefore, the aim of this study is to evaluate and compare the effectiveness of the KPIs xG and EPV in predicting match outcomes, in order to assess their value for soccer match analysis. Therefore, two distinct approaches to predict match outcomes are developed based on (i) pre-match information and (ii) post-match information.

## 2 Methods

This study was conducted according to the guidelines of the Declaration of Helsinki and approved by the local ethics committee (Human and Business Sciences Institute, Saarland University, Germany, identification number: 22-02, 10 January 2022).

### 2.1 Data

For this study official event and tracking data of a total of 918 matches of three consecutive Bundesliga seasons (German first division 2022/23, 2023/24, & 2024/25) were analyzed.

Sportec Solutions (Sportec Solutions AG, Ismaning, Germany) collected the notational event data live during the matches. Afterward it was subjected to post-match quality checks and includes about 30 different events with over 100 attributes, as defined by the official match data catalog of the German Soccer League (DFL) (22).

This data is supplemented by the tracking data (measuring frequency of 25 Hz) for both the ball and the on-field players. It was measured using a semi-automatic multi-camera tracking system (TRACAB, ChyronHego, Melville, NY, USA) which has been validated for soccer-specific performance assessment (23).

To effectively combine both data types they were synchronized (24).

All steps of data analysis, modeling, and visualization were completed using python 3.11.8 using the Pandas, NumPy, Math, SciPy, Matplotlib, SHAP, and scikit-learn libraries. To ensure the study's traceability, the main processing steps are outlined below. This includes the specification of models used in this study (xG, EPV, xBG), the detailed description of pre-match and post-match prediction approaches, and the description of the evaluation metrics employed to quantify the prediction performance.

To compare the prediction performance of xG and EPV, two independent and distinct approaches were developed for each model, applied to both pre-match and post-match scenarios resulting in 4 main prediction approaches (i.e., xG pre-match, EPV pre-match, xG post-match, EPV post-match).

### 2.2 xG/EPV/xBG

To predict match outcomes of soccer matches, the information of three different machine learning models were used. This includes expected goals (xG), expected possession value (EPV), and expected ball gain (xBG).

#### 2.2.1 xG

The outcome of xG quantifies the probability of a shot ending in a goal. There have been various approaches to model this prominent problem in soccer analytics, differing in the features used and their prediction performance. Overviews of different published xG models can be found in Cavus and Biecek (25) or Hewitt and Karakus (4). The xG model used in this study (24) demonstrated strong prediction performance which is shown in Table 1.

**Table 1:** Prediction performance of machine learning models expected goals (xG), expected possession value (EPV), and expected ball gain (xBG) on an unseen dataset of 306 matches of Bundesliga season 2024/25.

Description	Test set (306 matches of Bundesliga season 2023/24)					
	Accuracy	Recall	Precision	F1-Score	AUC	logloss
xG	0.90	0.24	0.71	0.36	0.81	0.28
EPV	0.96	0.36	0.09	0.14	0.79	0.17
xBG	0.81	0.13	0.23	0.17	0.61	0.50

#### 2.2.2 EPV

In contrast to xG, EPV quantifies the probability of scoring a goal in the following seconds of every match situation on the pitch (8, 26). Accordingly, it holds information about consecutive actions and allows to analyze whole possessions of an attacking team. Since every match situation in soccer is unique, the match analytical concept of playing phases helps to group similar match situations with comparable match situational context (e.g., offensive play vs. transition vs. set piece) (27, 28). The used EPV model of this study was trained individually for several distinct playing phases (i.e., offensive play, offensive transition, and 4 different set piece phases including direct freekicks, indirect freekicks, corners, and throw ins) (8). The features used to predict EPV (defined as the probability of scoring a goal within the following ten seconds of a given match situation) comprised 38 features capturing the match situation (e.g., distance to goal, relative pitch position), offensive performance (e.g., availability of passing options, deep runs), and defensive performance (e.g., defensive pressure, team organization). All detailed model descriptions can be accessed in (8). The prediction performance of the combined EPV model can be accessed in Table 1.

#### 2.2.3 xBG

To complement the offensive information about the scoring probability of every match situation for the attacking team, a defensive model was applied to quantify the probability of the defending team to regain the ball in every match situation (9, 29). xBG information can complement EPV information to quantify the risk and reward for both opposing teams of every single match situation. In detail, the attacking team's reward of scoring can be determined by the magnitude of EPV, while the attacking team's risk to lose ball possession can be evaluated by the size of the opposing xBG (8). Table 1 indicates the prediction performance of the xBG model which was developed using a similar playing phase based approach to EPV.

### 2.3 Pre-match predictions

To predict the match outcome based on pre-match information a two-stage approach was developed. In the first stage, machine learning models were developed to predict the number of goals scored for both teams. Those models were trained solely on information of the match performances of both opposing teams in the last three matches. In the second stage, this predicted number of goals for both teams were used to simulate the match outcome to quantify the probabilities.

#### 2.3.1 Features

To effectively predict the target variable of number of goals scored by an individual team (i.e., considered team) in an upcoming match (against an upcoming opponent), we engineered features based on the core idea introduced by Dixon and Coles for forecasting future performances (15).

Thereby, a well-designed model for predicting match outcomes should incorporate the following aspects: (i) the abilities of both teams, (ii) the contextual factor of match venue (home vs. away), (iii) a team's ability should be reflected by its recent performances, (iv) this ability should consider both offensive and defensive strengths, and (v) those recent performances should be weighted by the strength of the opponents faced in those matches.

Therefore, the following features were computed, consisting of information about the match context (e.g., venue), the relative quality of the opponent (e.g., difference in table position between considered and opposing team), the attacking performance of the considered team in the last three matches (e.g., EPV measures, xG measures), defensive performance of the upcoming opponent in the last three matches (e.g., xBG measures, xG conceded measures), and features describing the difficulty of the last three matches for both the considered team and the upcoming opponent (e.g., table position of the last three opponents).

All detailed feature specifications and their description can be found in Table 2.

**Table 2:** Features used to predict the number of goals scored by the considered team in an upcoming match against an upcoming opponent.

Category	Subcategory	Feature	Exact feature expression	Description
Match context		match_venue	Categorical: Home (≙0), Away (≙1)	Match venue of the considered match.
Opponent strength	Table position	difference_table_position	Ordinal: (−17–17)	Difference in table position (1–18) between the considered team and the upcoming opponent throughout the season until the considered match.
		difference_table_position_last3_0	Ordinal: (−17–17)	Difference in table position (1–18) between the considered team and the upcoming opponent throughout the last 3 matches.
	Points gained	difference_points_mean	Numerical: (−3–3)	Difference in points gained between the considered team and the upcoming opponent throughout the season until the considered match, normalized on the matches played.
		difference_points_last3_0	Numerical: (−9–9)	Difference in points gained between the considered team and the upcoming opponent throughout the last 3 matches.
Match difficulty of last 3 matches	Considered team	difference_table_position_last3	Ordinal: (−17–17)	Difference in table position between the considered team and each of its last 3 opponents (considering the table of the last 3 matchdays at the time the match was played).
		difference_points_last3	Numerical: (−3–3)	Difference in points gained between the considered team and each of its last 3 opponents (considering the last 3 matches at the time the match was played).
	Opposing team	opp_difference_table_position_last3	Ordinal: (−17–17)	Difference in table position between the upcoming opponent and each of its last 3 opponents (considering the table of the last 3 matchdays at the time the match was played).
		opp_difference_points_last3	Numerical: (−3–3)	Difference in points gained between the upcoming opponent and each of its last 3 opponents (considering the last 3 matches at the time the match was played).
Offensive performance of considered team (shots & goals)	Goals	goals_last_3	Ordinal	Number of goals scored by the considered team in the last 3 matches.
		goal_difference_last_3	Ordinal	Goal difference of the considered team in the last 3 matches.
	Shots	shots_last_3	Ordinal	Number of shots by the considered team in the last 3 matches.
Offensive performance of considered team (EPV & opposing xBG)	EPV	epv_last_3	Numerical	Sum of EPV gained (computed as the maximum of each possession of the considered team) by the considered team in the last 3 matches.
		epv_over50_last_3	Numerical	Sum of possessions by the considered team where EPV exceeded 0.5 in the last 3 matches.
	EPV & xBG	epv_comp_last_3	Numerical	Sum of EPV-xBG gained (computed as the maximum of each possession of the considered team) by the considered team in the last 3 matches.
		epv_comp_over50_last_3	Numerical	Sum of possessions by the considered team where EPV-xBG exceeded 0.5 in the last 3 matches
	EPV post-match pred	pred_goals_epv_last_3	Numerical	Number of predicted goals by the considered team in the last 3 matches based on EPV post-match prediction.
		pred_goal_difference_epv_last_3	Numerical	Predicted goal difference by the considered team in the last 3 matches based on EPV post-match prediction.
		pred_points_epv_last_3	Numerical	Predicted number of points gained by the considered team in the last 3 matches based on EPV post-match prediction.
	Performance vs. EPV post-match pred	performance_pred_goals_epv_last_3	Numerical	Difference in actual and predicted number of goals scored by the considered team in the last 3 matches based on EPV post-match prediction.
		performance_pred_points_epv_last_3	Numerical	Difference in actual and predicted number of points gained by the considered team in the last 3 matches based on EPV post-match prediction.
		performance_pred_goal_difference_epv_last_3	Numerical	Difference in actual and predicted goal difference by the considered team in the last 3 matches based on EPV post-match prediction.
Offensive performance of considered team (xG)	xG	xg_last_3	Numerical	Sum of xG gained by the considered team in the last 3 matches.
	xG post-match pred	pred_goals_xg_last_3	Numerical	Number of predicted goals by the considered team in the last 3 matches based on xG post-match prediction.
		pred_points_xg_last_3	Numerical	Predicted goal difference by the considered team in the last 3 matches based on xG post-match prediction.
		pred_goal_difference_xg_last_3	Numerical	Predicted number of points gained by the considered team in the last 3 matches based on xG post-match prediction.
	Performance vs. xG post-match pred	performance_pred_goals_xg_last_3	Numerical	Difference in actual and predicted number of goals scored by the considered team in the last 3 matches based on xG post-match prediction.
		performance_pred_points_xg_last_3	Numerical	Difference in actual and predicted number of points gained by the considered team in the last 3 matches based on xG post-match prediction.
		performance_pred_goal_difference_xg_last_3	Numerical	Difference in actual and predicted goal difference by the considered team in the last 3 matches based on xG post-match prediction.
Defensive performance of upcoming opponent (conceded shots & goals)	Conceded goals	opp_conceded_goals_last_3	Numerical	Number of goals conceded by the upcoming opponent in the last 3 matches.
		opp_conceded_goal_difference_last_3	Numerical	Goal difference by the upcoming opponent in the last 3 matches.
	Conceded shots	opp_conceded_shots_last_3	Numerical	Number of shots conceded by the upcoming opponent in the last 3 matches.
Defensive performance of upcoming opponent (xBG & conceded EPV)	xBG	opp_xbg_last_3	Numerical	Sum of xBG gained (computed as the maximum of each possession by the upcoming opponent) by the upcoming opponent in the last 3 matches.
		opp_xbg_over50_last_3	Numerical	Sum of possessions by the upcoming opponent where xBG exceeded 0.5 in the last 3 matches.
	EPV conceded	opp_conceded_epv_last_3	Numerical	Sum of EPV conceded (computed as the maximum of each possession of the opponents of the upcoming opponent) by the upcoming opponent team in the last 3 matches.
		opp_conceded_epv_over50_last_3	Numerical	Sum of possessions by the opponents of the upcoming opponent where EPV exceeded 0.5 in the last 3 matches.
	EPV post-match pred	opp_pred_conceded_goals_epv_last_3	Numerical	Number of predicted goals conceded by the upcoming opponent in the last 3 matches based on EPV post-match prediction.
		opp_pred_conceded_goal_difference_epv_last_3	Numerical	Predicted goal difference by the upcoming opponent in the last 3 matches based on EPV post-match prediction.
		opp_pred_points_epv_last_3	Numerical	Predicted number of points gained by the upcoming opponent in the last 3 matches based on EPV post-match prediction.
	Performance vs. EPV post-match pred	performance_opp_pred_conceded_goals_epv_last_3	Numerical	Difference in actual and predicted number of goals conceded by the upcoming opponent in the last 3 matches based on EPV post-match prediction.
		performance_opp_pred_points_epv_last_3	Numerical	Difference in actual and predicted number of points gained by the upcoming opponent in the last 3 matches based on EPV post-match prediction.
		performance_opp_pred_conceded_goal_difference_epv_last_3	Numerical	Difference in actual and predicted goal difference by the upcoming opponent in the last 3 matches based on EPV post-match prediction.
Defensive performance of upcoming opponent (conceded xG)	xG conceded	opp_conceded_xg_last_3	Numerical	Sum of xG conceded by the upcoming opponent in the last 3 matches.
	xG post-match pred	opp_pred_conceded_goals_xg_last_3	Numerical	Number of predicted goals conceded by the upcoming opponent in the last 3 matches based on xG post-match prediction.
		opp_pred_points_xg_last_3	Numerical	Predicted goal difference by the upcoming opponent in the last 3 matches based on xG post-match prediction.
		opp_pred_conceded_goal_difference_xg_last_3	Numerical	Predicted number of points gained by the upcoming opponent in the last 3 matches based on xG post-match prediction.
	Performance vs. xG post-match pred	performance_opp_pred_conceded_goals_xg_last_3	Numerical	Difference in actual and predicted number of goals conceded by the upcoming opponent in the last 3 matches based on xG post-match prediction.
		performance_opp_pred_points_xg_last_3	Numerical	Difference in actual and predicted number of points gained by the upcoming opponent in the last 3 matches based on xG post-match prediction.
		performance_opp_pred_conceded_goal_difference_xg_last_3	Numerical	Difference in actual and predicted goal difference by the upcoming opponent in the last 3 matches based on xG post-match prediction.

Features are categorized into match context, strength of opponent, match difficulty of last three matches, offensive performance of considered team, and defensive performance of upcoming opponent. The features related to number of shots and goals are colored in green, the features related to EPV and xBG are colored in orange, and the features based on xG are colored in blue.

#### 2.3.2 Modelling of number of goals (xGoalNumber)

The prediction approach to predict the number of goals of a considered team in an upcoming match against an upcoming opponent (xGoalNumber) used information of the match performance of the last three matches of each team. However, to assess the difficulty of each team's third-to-last opponent, the table positions prior to that match were calculated based on the last three matches.

To ensure sufficient performance data of the competing teams the first six matchdays of both seasons were excluded. Therefore, a total of 504 matches with 1,008 case samples of individual teams were included in the prediction approach.

Random Forest and XGBoost regression models were trained for both the xG pre-match and EPV pre-match approaches. For the EPV-pre match approach, all xG-related variables were excluded (see blue features in Table 2), ensuring the approach only incorporates information about context, strength of opponent, offensive and defensive performance of shots, goals, EPV, and xBG. Conversely, for the xG approach, all EPV- and xBG-related variables were excluded (see orange features in Table 2).

An 80/20 hold-out split was applied on a match-by-match basis to prevent data leakage by ensuring that no samples from the same match appeared in both the training and test datasets. With the 80% training dataset a five-fold cross validation with hyperparameter tuning on a randomized grid search and performance optimization on RMSE was applied.

For the selected best performing models, SHAP values and feature importances were examined. SHAP values, which are based on cooperative game theory, quantify the contribution of each feature to a model's output (30). In doing so, they provide insights both into the overall importance of each feature for predictions and into how specific feature values influence individual predictions. This allows for a more comprehensive and detailed interpretation of the presented machine learning models.

#### 2.3.3 Prediction of match outcomes

After the prediction, a double Poisson distribution (with a maximum of eight goals scored in the current dataset) was applied to the absolute predicted number of goals for each team estimated by the machine learning model. Accordingly, the home and away team scores are modeled as independent Poisson distributions, following Maher (31). The approach to model match outcomes based on Poisson distributions was used in line with (15, 32, 33) which indicated substantial predictive performance. This distribution was then used to simulate the match outcome 10,000 times.

Similar to the post-match approach those results were used afterwards to quantify the probabilities of the final match outcome.

#### 2.3.4 Baseline approach

To establish a benchmark for evaluating the presented pre-match prediction models, we implemented an Elo-based match prediction as a baseline (34). Specifically, the approach models the outcome probabilities of home win, draw, and away win using the Bradley–Terry–Davidson framework to account for draws (35, 36), incorporating an average draw rate of 0.25 derived from the Bundesliga 2022/23 and 2023/24 seasons. Further, a fixed home advantage of 60 Elo points and a k-factor of 20 were applied to adjust ratings after each match, starting from a uniform base Elo of 1,500 for all teams. This baseline provides a simple yet interpretable framework for comparison, ensuring that the presented pre-match prediction models can be assessed against a standardized and widely recognized predictive method.

### 2.4 Post-match predictions

To predict the match outcome based on post-match information, two approaches—xG and EPV—were applied for both teams after the considered match. For each match, 10,000 match simulations were completed. Each match simulation predicted whether a goal would result on each individual shot (xG—post match) or each individual possession (EPV—post match) based on the computed probabilities of the considered models.

This process generated 10,000 potential match outcomes with regard of the performance of both teams during the match. The distribution of the match outcomes was then used to quantify the probabilities of the final match outcome.

### 2.5 Evaluation

To evaluate and compare the EPV and xG post-game and pre-game approaches the ranked probability score (RPS), the accuracy of the probabilistic forecasts of the match results, and the Brier Score (including its decomposition into uncertainty, reliability, & resolution) were employed. Those performance metrics were chosen as they are standard proper scoring rules for evaluating probabilistic forecasts of multi-class outcomes such as win/draw/loss (13, 18).

RPS evaluates the accuracy of predicted probabilities by comparing the forecasted probabilities for all possible match outcomes (home win, draw, away win) against the actual outcome (18, 37). In detail, if a model predicts probabilities of 0.6 for a home win, 0.3 for a draw, and 0.1 for an away win, and the actual outcome is a draw, the RPS is calculated by summing the squared differences between the cumulative predicted probabilities [=(0.6, 0.9, 1.0)] and the cumulative observed probabilities [=(0, 1, 1)] across the three outcomes and dividing by the number of categories minus one (i.e., RPS = ½[(0.6–0)² + (0.9–1)² + (1.0–1)²] = ½(0.36 + 0.01 + 0) = 0.185).

The accuracy was defined by the proportion of samples where the predicted outcome (home win, draw, away win) with the highest probability matches the actual outcome of the match. In detail, given predicted probabilities of 0.6 for a home win, 0.3 for a draw, and 0.1 for an away win, a home win would be counted as correct, while a draw or away win would be counted as incorrect.

The Brier Score measures the mean squared difference between predicted probabilities and actual outcomes. In detail, given the presented example above (probabilities: 0.6 home win, 0.3 draw, 0.1 away win) and an actual result of a draw, the Brier score is calculated as follows: Brier Score = ⅓[(0.6–0)² + (0.3–1)² + (0.1–0)²] = 0.287. Furthermore, the Brier Score can be decomposed into uncertainty, reliability, and resolution. Uncertainty reflects the inherent variability of outcomes, reliability measures the calibration of predicted probabilities, and resolution evaluates the ability of the forecasts to discriminate between different outcome frequencies.

For the modeling of number of goals of an upcoming match the following evaluation metrics were computed: Mean absolute error (MAE), Mean Squared Error (MSE), root mean squared error (RMSE), and R2.

## 3 Results

Of the 756 matches analyzed for the match outcome prediction [28 matchdays (matchday 7–34) in all three analyzed seasons], home teams won in 335 encounters (44.3%), while 195 matches ended in a draw (25.8%), and away teams secured 226 wins (29.9%). On average, home teams scored 1.79 ± 1.45 goals per match, totaling 1,351 goals, whereas away teams recorded 1.36 ± 1.19 goals per match, amounting to 1,026 goals.

The most frequently observed match result was a 1:1 draw (12.3%), followed by home wins of 2:1 (7.5%) and 2:0 (7.1%), and a 1:2 (6.5%) win on the road. The match with the most scored goals ended 8:1 and the match with the highest goal differences was an 8:0 home win.

**Figure 1:**
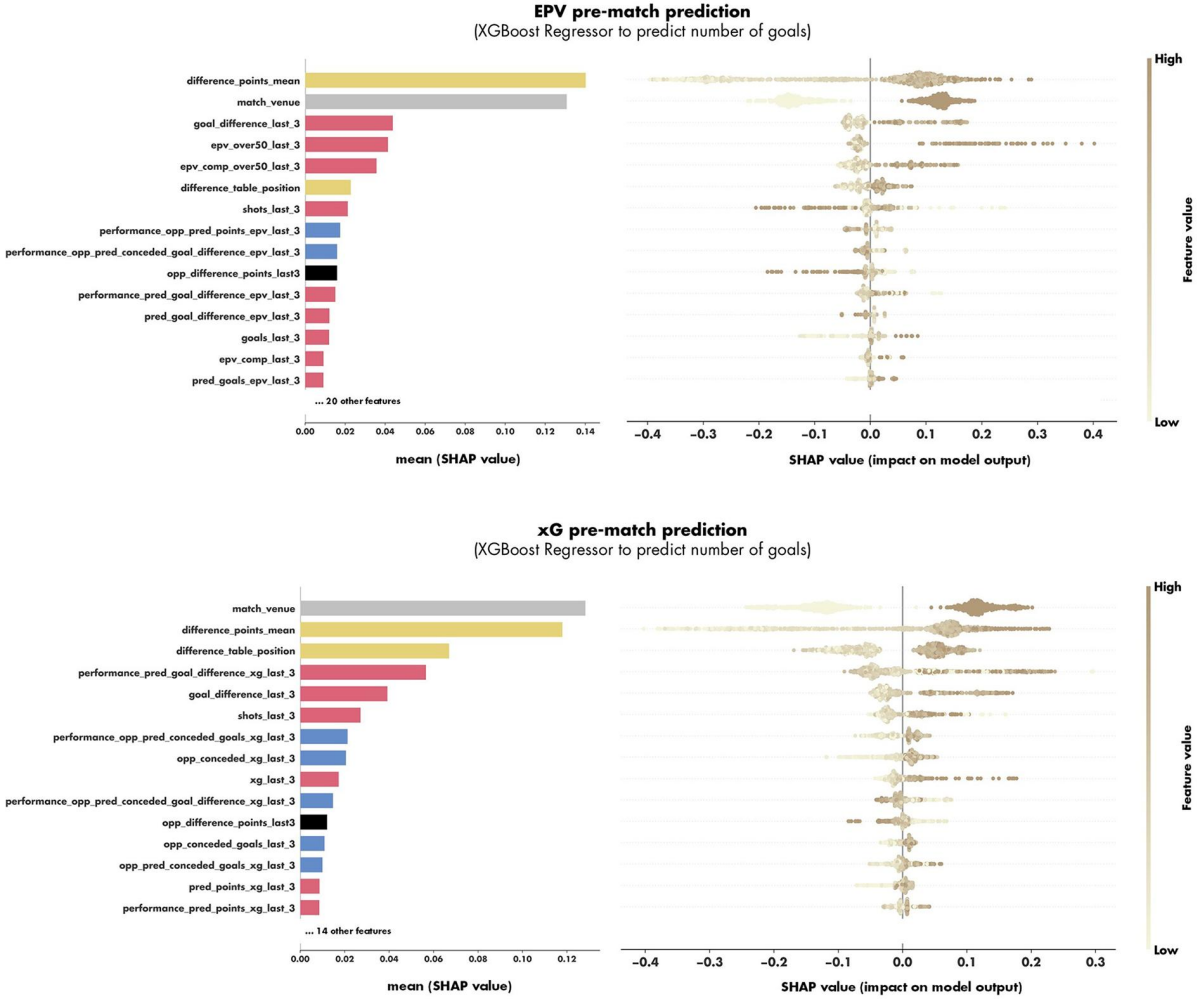
The feature importance (on the left) and SHAP values (on the right) of the features included in the XGBoost models of the models predicting the number of goals scored in an upcoming match against an upcoming opponent based on EPV information (at the top) and xG information (at the bottom). The features are colored according to their category (match context: grey, opponent strength: black, match difficulty: yellow, offensive performance considered team: red, defensive performance of upcoming opponent: blue).

**Figure 2:**
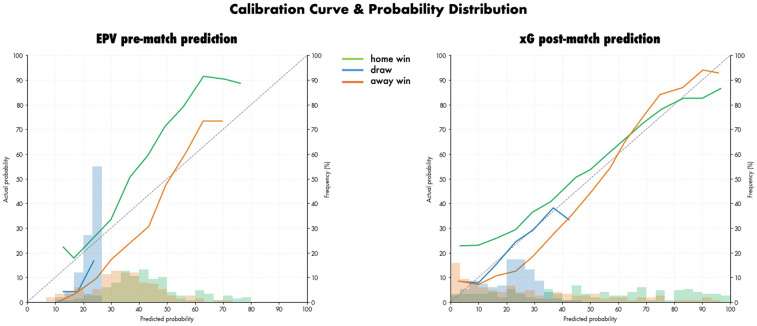
Calibration curves and probability distributions of match outcome predictions for the EPV pre-match approach (left) and the xG post-match approach (right). Home wins are shown in green, away wins in blue, and draws in grey.

For the pre-match prediction approaches, the xGoalNumber models showed satisfactory prediction performance for both EPV and xG information scenarios (see Table 3). For EPV information the XGBoost Regressor showed increased prediction performance of the number of goals (MSE) and in the pre-match predictions of the match outcome (RPS & Accuracy) compared to Random Forest Regressors. Overall, the XGBoost Regressor on the EPV pre-match approach showed the best pre-match outcome prediction performance (RPS: 0.194, Accuracy: 0.583). The SHAP values of the xGoalNumber models for EPV and xG (XGBoost Regressors) are displayed in Figure 1. Furthermore, the calibration and probability distribution of the selected EPV pre-match and xG post-match models are illustrated in Figure 2.

**Table 3:** Prediction performance of pre-match prediction approaches at the top (including information about the prediction performance of models to predict the number of goals in an upcoming match) and post-match prediction approaches at the bottom.

xGoalNumber											Pre-match prediction		
Description	Information		Training Set (80%)				Test Set (20%)				Test Set (20%)		
			performance in 5-fold cross validation (Ø mean & ± standard deviation) [95% confidence intervals]										
	Number of features	Algorithm	MAE	MSE	RMSE	R2	MAE	MSE	RMSE	R2	RPS	Accuracy	Brier Score (REL; RES; UNC)
xG-pre match (last 3)	29	XGBoostRegressor	1,007 (Ø 1,007 ± 0,066) [1,089, 0,925]	1,615 (Ø 1,615 ± 0,241) [1,914, 1,316]	1,268 (Ø 1,268 ± 0,091) [1,382, 1,155]	0,080 (Ø 0,080 ± 0,019) [0,056, 0,105]	1,038	1,779	1,334	0,098	0,197	0,537	0,188 (0,014; 0,026; 0,200)
xG-pre match (last 3)	29	RandomForestRegressor	1,025 (Ø 1,025 ± 0,049) [1,086, 0,964]	1,689 (Ø 1,689 ± 0,153) [1,879, 1,500]	1,299 (Ø 1,299 ± 0,060) [1,373, 1,224]	0,092 (Ø 0,092 ± 0,027) [0,058, 0,125]	**0,934**	**1,398**	**1,182**	0,112	0,199	0,556	0,189 (0,023; 0,024; 0,200)
EPV-pre match (last 3)	35	XGBoostRegressor	0,996 (Ø 0,996 ± 0,068) [1,081, 0,912]	1,582 (Ø 1,582 ± 0,221) [1,857, 1,307]	1,255 (Ø 1,255 ± 0,085) [1,361, 1,150]	0,098 (Ø 0,098 ± 0,029) [0,062, 0,135]	1,021	1,711	1,308	**0,132**	**0,194**	**0,583**	**0,185 (0,022; 0,037; 0,200)**
EPV-pre match (last 3)	35	RandomForestRegressor	1,021 (Ø 1,021 ± 0,038) [1,068, 0,974]	1,649 (Ø 1,649 ± 0,116) [1,793, 1,506]	1,284 (Ø 1,284 ± 0,045) [1,340, 1,227]	0,111 (Ø 0,111 ± 0,037) [0,065, 0,158]	0,940	1,409	1,187	0,105	0,197	0,536	0,187 (0,018; 0,029; 0,200)
Elo rating											0,202	0,553	0,187 (0,013; 0,024; 0,200)
Post-match prediction													
Description	Information							Overall (100%)			Test Set (20%)		
	Number of features	Algorithm						RPS	Accuracy	Brier score (REL; RES; UNC)	RPS	Accuracy	Brier score (REL; RES; UNC)
xG-post match	1	10.000 simulations						0,164	0,604	0,168 (0,002; 0,050; 0,217)	**0,148**	**0,656**	**0,151 (0,004; 0,054; 0,200)**
EPV-post match	1	10.000 simulations						0,203	0,531	0,203 (0,011; 0,026; 0,217)	0,191	0,596	0,182 (0,017; 0,035; 0,200)

The performance measures of ranked probability score (RPS), accuracy and brier score on the 20% hold out test set with the best predictive performances highlighted in bold.

For post-match prediction, the xG approach showed the best performance (RPS: 0.148, Accuracy: 0.656) and thus demonstrated better performance compared to all pre-match approaches. The EPV post-match approach showed worse prediction performance compared to xG information of the played match (RPS: 0.191, Accuracy: 0.596).

## 4 Discussion

This study aimed to evaluate and compare the effectiveness of the KPIs expected goals (xG) and expected possession value (EPV) in predicting match outcomes, in order to assess their value for soccer match analysis. With it, two main approaches were developed to predict match outcomes, a pre-match approach processing information about the latest match performances of the two opponents, and a post-match approach containing information about the performance of both teams in the played match.

Overall, the results indicated that the pre-match approaches using EPV information showed increased prediction performance (RPS = 0.194, Accuracy = 0.583) compared to xG information (RPS = 0.199, Accuracy = 0.556). Thereby, the presented pre-match approaches outperformed the baseline Elo rating model (RPS = 0.202, Accuracy = 0.553), which still demonstrated robust predictive performance. This indicates that the contextual information of match venue and team strength in combination with the information of the performance of the last three matches hold sufficient information to effectively predict future match outcomes in the Bundesliga. In contrast, the post-match approaches including xG information outperformed EPV information.

In detail, the xG post-match approach showed the best prediction performance of the match outcome of all approaches made in this study (RPS = 0.148, Accuracy = 0.656) and thereby showed increased performance compared to the EPV post-match approach (RPS = 0.191, Accuracy = 0.596). This may be traced back to the fact that in soccer shots are practically the only way to score (except from own goals). This result is in line with previous studies on goal scoring in international tournaments (38, 39), which indicate that both the number of shots and their quality (e.g., shots on target) are decisive success factors. Therefore, the reduced information of the chances created in a match that led to shots at goal holds highly objective information on the match performance of a team which is underlined by the presented results. Therefore, the information on the amount and magnitude of goal scoring chances is a highly powerful information to predict match outcomes after the match is played. With it, the xG performance of a team is a highly objective measure of a team's offensive performance, helping to filter out chance in match outcomes and provide a more accurate evaluation of team performance.

While post-match approaches benefit from the reduced information of offensive performance in the reduction of shots, they overlook a significant amount of attacking play that does not led to shots at goal (e.g., chances without shots). In this case, EPV appears to hold more detailed information on the scoring probability encountering from every action and thus every attacking sequence of a team. EPV may therefore provide a more comprehensive representation of a team's scoring potential of an entire attack even in the absence of a shot and a resulting xG value. This may be one of the reasons why the EPV pre-match prediction (RPS = 0.194, Accuracy = 0.583) slightly outperformed the xG pre-match approach (RPS = 0.199, Accuracy = 0.556) in predicting the outcome of an upcoming match. Thereby, the reductionist information on the scoring probability of shots may hold fewer information on the overall performance of a team.

In addition to comparing the prediction performance of the different approaches, gaining insights into the most important features for the pre-match prediction is of interest. The most important features in predicting the number of goals in an upcoming match were features of the opponent's strength (difference points mean 1st in EPV, 2nd in xG; difference table position 6th in EPV, 3rd in xG). In detail, the SHAP values indicate that more goals are predicted if the considered team has more points and is ranked higher in the table of the current season compared to the upcoming opponent. Furthermore, the model assigns greater importance to the number of points than to table position, likely because points provide a more granular and informative signal of team performance relevant to goal prediction.

Besides the opponent strength, the match venue contained highly important information for the prediction approaches (1st in xG, 2nd in EPV) indicating that home teams are predicted to score a higher number of goals compared to teams on the road. This home advantage is supported by several findings in the literature (40).

The offensive performance of the considered team was represented most importantly by the goal difference achieved (3rd in EPV, 5th in xG) and the number of shots taken (7th in EPV, 6th in xG) in the last three matches. In contrast, the raw number of goals of last three matches showed few predictive power (13th in EPV, and not under the best 15th in xG). This suggests that goals may involve a significant element of chance (24), making them less reliable for predicting future performance and match outcomes.

In addition to offensive metrics (i.e., number of shots and goals), the detailed KPIs of EPV and xG also indicated a high importance in predictions. In detail, EPV (triggered over 0.5) in the last three matches (4th) showed the highest importance of all EPV features in the EPV pre-match approach. This KPI reflects the number of possessions in which the EPV model predicts a goal, highlighting its high value for match analysis. In contrast, the sum of xG of the last three matches (9th) was the most important xG feature in the xG pre-match approach. Once again, this finding underscores that xG provides limited value in predicting the number of goals or match outcomes in the future compared to EPV.

While offensive features demonstrated strong predictive power, features capturing the defensive performance of the upcoming opponent still provided meaningful contributions to the prediction models (e.g., conceded xG & goals in the last three matches). This highlights the importance of incorporating defensive metrics into tactical match analysis which has been underrepresented in the recent literature (9, 28).

In the end, the match difficulty of the last three matches of the upcoming opponent was also of certain important for the predictions (10th in EPV, 11th in xG). The SHAP values indicated that when the upcoming opponent faced weaker teams in their previous three matches, a higher number of goals was predicted for the considered team. This effect may be explained by the potential momentum gained from favorable outcomes against weaker opponents, which could positively influence a teams upcoming performance.

While analyzing individual features is important for enhancing interpretability and identifying key information, the true relevance of the predictions lies in evaluating their overall predictive performance. Therefore, the prediction performance of the presented models is discussed in the light of comparable studies. While the EPV pre-match approach showed the best pre-match prediction performance (RPS = 0.194, Accuracy = 0.583) the xG post-match approach showed the best post-match prediction performance (RPS = 0.148, Accuracy = 0.656). Thereby, the presented approaches slightly outperformed the approaches developed by Berrar et al. (18) (RPS: 0.202, Accuracy: 0.519) which incorporated expert-knowledge to predict match outcomes in the 2017 soccer prediction challenge (dataset of 52 leagues). Still the approaches presented by Berrar et al. (18) clearly outperformed all approaches developed and tested in the summary work by Hubáček et al. (13) (best RPS: 0.210, best Accuracy: 0.486) and all approaches developed and evaluated in the soccer prediction challenge 2023 (best model RPS: 0.211) (12). Additionally, the prediction performance of the betting market odds were presented with an RPS of 0.206 (12). While the approaches presented by Berrar et al. (18) incorporated similar feature groups compared to the presented study (i.e., attacking performance, defensive performance, recent performance, strength of opposition, home advantage), they solely relied on event data. This considerably effects the granularity of the performance features (24) and, consequently, the predictive capability of the developed models.

Given such methodological differences, such comparisons between different match outcome prediction studies in the literature must be approached with caution. The datasets used in these studies vary not only in terms of data types (e.g., event data vs. tracking data), which mainly differ in the level of detail provided, but also in the kinds of information included. Thereby, features included range from past team performances (e.g., performances from the last three matches up to entire seasons) to contextual factors such as match venue, coach replacements, or player injuries. Additionally, many studies use data from different leagues and seasons, which directly influences the results and limits comparability. However, one of the most critical aspects when comparing prediction models is the scope of their application. While some approaches are designed to forecast outcomes over entire seasons or across multiple matchdays, the present model is tailored to predict only the upcoming matchday of a single competition, allowing for continuous updates based on the most recent data. Despite the inherent limitations in comparability, such evaluations provide valuable insights into the relative strengths and practical applicability of different prediction approaches.

Building on these distinctions, the presented approach offers significant advantages by encompassing a wide spectrum of prediction factors (systematic and unsystematic effects) (14) and integrating domain knowledge which has been identified as key quality in the modelling process (18). Thereby, it integrates systematic effects, such as team-specific metrics like recent offensive performance, alongside global factors like home advantage. Additionally, it accounts for unsystematic elements, including random variability in match outcomes using double Poisson distributions. This comprehensive framework improves the model's adaptability and predictive accuracy.

While the present study focused on the domestic competition of Bundesliga and employed several probabilistic multi-class forecasting approaches for match outcomes, recent research on major tournaments such as the FIFA World Cup or the UEFA European Championship (38, 39, 41, 42) has primarily examined goal-scoring behavior, tactical determinants, and contextual match characteristics, often within binary classification frameworks (e.g., win vs. non-win). For instance, these analyses demonstrated that scoring the first goal substantially increases the likelihood of winning (38). While such studies provide valuable insights into the determinants of success, their findings are not directly comparable to the probabilistic modeling framework applied in the present work.

### 4.1 Practical application

**Figure 3:**
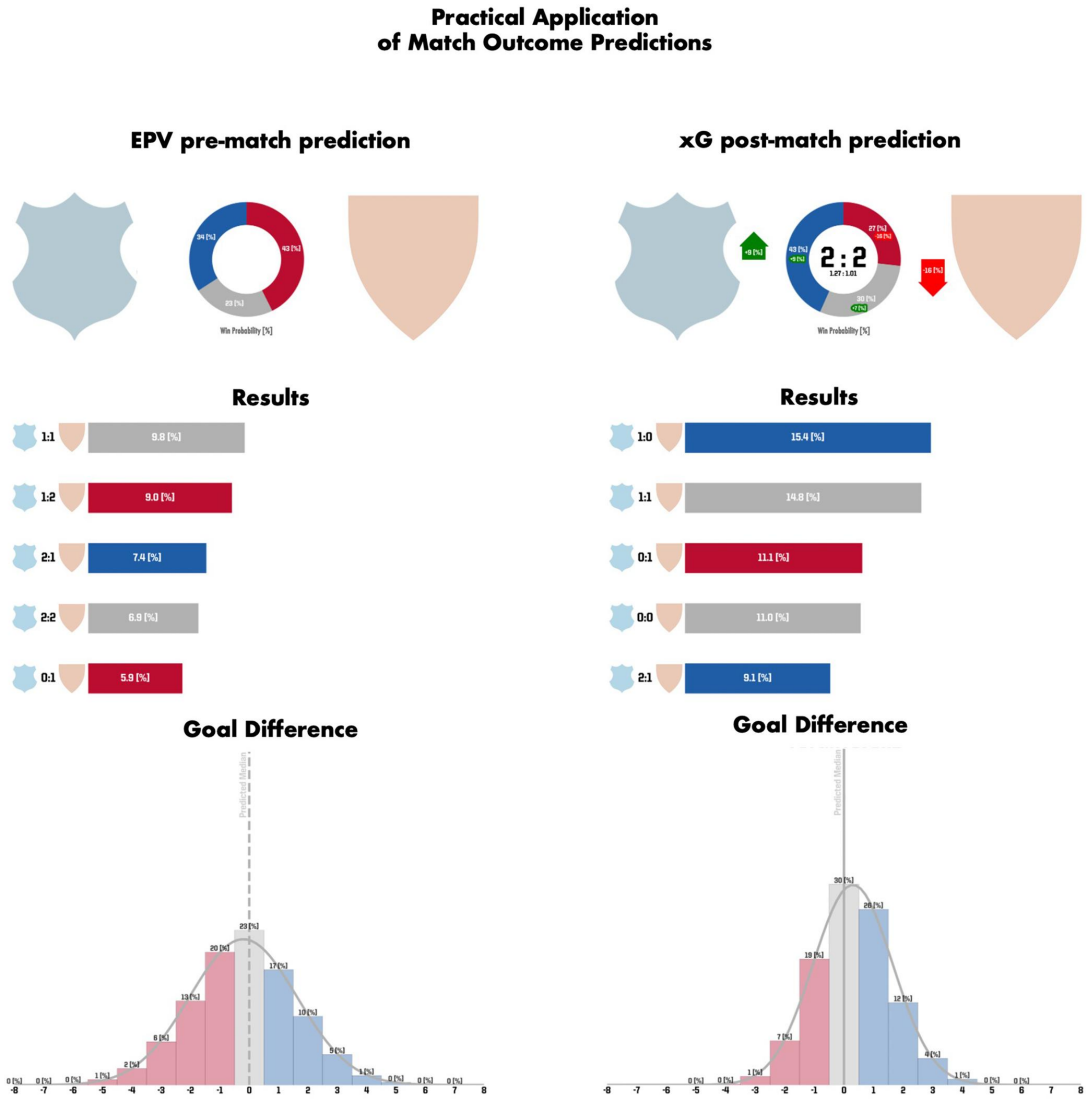
Both the pre-match prediction based on the EPV approach (on the left) and the post-match prediction based on the xG approach (on the right) are depicted for an exemplary match analysis. At the top, the outcome probabilities of home win (blue), draw (grey) and away win (red) are illustrated. With it, the differences between the pre-match and post-match predictions as well as the final result and the xG values for both teams are depicted at the right top. Specifically, the home team increased their win probability by 9% (and reduced their loss probability by 16 %) by outperforming the opponent in expected goals (xG: 1.27 vs. 1.01). Still, the match ultimately ended in a 2:2 draw. In the middle, the five likeliest end results and their respective probabilities are specified. At the bottom, the predicted goal difference is visualized from the perspective of the home team. Thereby, a negative goal difference (indicating a predicted home loss) is represented in red, a positive goal difference (indicating a predicted home win) is represented in blue, and a goal difference of zero (indicating a predicted draw) is represented in grey.

Beyond the predictive performance of the presented approaches and the insights presented on the predictiveness of EPV and xG, the proposed approaches hold relevant practical value when used in real-case analysis scenarios. Thereby, the different pre-match and post-match approaches can be used in pre-match (e.g., opponent analysis), and post-match analyses (e.g., own team analysis) as well as supporting seasonal analyses. In detail, pre-match approaches can effectively be applied in pre-match preparations of a club to estimate a team's chances of winning. Furthermore, they can be used to predict the final or winter break table ranking results of a season which can be used to inform decision-making based on the current performance trend of a team. Additionally, the post-match approaches can be used in post-match analyses to offer an objective evaluation of in-game performances. However, the key insights arise when both approaches can be compared simultaneously thereby, measuring the objective expectations against the actual performance and the final result. Thereby, analyzing the shift between pre- and post-match predictions provides valuable insights for coaching staff, highlighting changes in winning probabilities and supporting data-driven performance assessments. This use case is exemplarily depicted in Figure 3.

Moreover, the applied SHAP analysis (see Figure 1) can also be used to decompose individual predictions into their contributing factors (43). By implementing such instance-level SHAP analyses, analysts and coaching staff can identify which variables most strongly influenced specific match outcome predictions (in both pre-match and post-match scenarios). This further enhances the practical applicability of the presented models by increasing their interpretability.

### 4.2 Limitations and future research

While the results indicate substantial prediction performance of the presented approaches, the presented study still comes with limitations that have to be noticed when interpreting the results.

First, the prediction models presented did not incorporate information on individual players, such as the specific offensive and defensive performance of players on the pitch such as individual contributions to EPV or xG created or conceded. Including such individual player information (e.g., by analyzing the starting line-up of both teams) could potentially enhance the model's predictive power, as changes in player availability due to injuries, suspensions, or tactical decisions can significantly impact match outcomes (44).

Second, the pre-match prediction approaches solely included an approach using a double Poisson distribution based on the predicted number of goals scored in an upcoming match (32). While this distribution was chosen as it has been shown to deliver robust results (13) it comes with the risk of underestimating draws, even though a draw of 1:1 was the most frequent result in the current sample (13.7% in the presented sample, see results). Future studies should therefore analyze the effects of different distributions on the accuracy of the predictions. For instance, more advanced modeling frameworks could be explored, such as Bayesian hierarchical models that account for uncertainty and team-specific effects, or temporal neural networks (e.g., recurrent or attention-based architectures) that capture the dynamic evolution of team performance over time (e.g., season). Such approaches may better integrate contextual and temporal dependencies, thereby potentially improving predictive accuracy, for instance by capturing latent team strength variations and match-to-match dependencies.

Additionally, the models only used match venue as contextual information. Other context features such as specific information of injuries, coaches, and other situational factors (e.g., period of the season) could be tested in future approaches.

Third, the presented analysis was based on a limited sample of three consecutive Bundesliga seasons (2022/23, 2023/24, & 2024/25). This limits insights into the temporal generalizability of the findings (e.g., across future seasons) and their transferability to other competitions (e.g., other leagues or cup competitions). Future research should therefore examine whether the proposed models or relationships remain stable across extended time spans and different competitive contexts.

Fourth, the pre-match predictions presented in this study were developed to predict the match outcomes of the following matchday. Besides, future studies could analyze the predictive power of the presented approach over multiple upcoming matchdays or the remainder of the season.

Finally, a general limitation of machine learning-based prediction models lies in the interpretation of results. In contrast to classical statistical approaches, no universally established thresholds (e.g., *p*-values or effect sizes) are available. Consequently, interpretability may be constrained in situations where feature importance values are of similar magnitude.

## 5 Conclusion

Overall, this study presented pre-match and post-match approaches to predict the match outcome of upcoming matches in the Bundesliga. Thereby, this study compared the predictiveness of the KPIs EPV and xG holding highly objective information on the match performance of teams. The results indicated that EPV is beneficial compared to xG in pre-match scenarios indicated by an increased prediction performance in predicting the outcome of an upcoming match. In contrast, xG outperformed EPV in post-match scenarios indicating its predictive power as highly objective measure of match performance. In conclusion, this study showcased the use of both approaches in match analysis settings thereby indicating that the combined analysis of pre- and post-match predictions holds highly important and useful information when objectively assessing team performances in elite soccer.

## Data Availability

The data analyzed in this study is subject to the following licenses/restrictions: The used data is property of the German Football League (Deutsche Fußball Liga, DFL) and is not publicly available. The authors do not have permission to share the data publicly. This work can be reproduced using similar data from professional soccer (e.g. tracking and event data of other soccer leagues). Requests to access these datasets should be directed to https://www.dfl.de/en/. The materials and code developed for the presented analysis are openly accessible at https://github.com/LForcher/ai_match_analysis. The repository provides detailed information on the data preprocessing procedures (including feature calculation), as well as the modeling pipelines used to train and evaluate the predictive models. While the used data is not publicly available, the available scripts allow replication of key parts of the analysis, including data preprocessing and modeling.

## References

[1] Reep C Benjamin B. Skill and chance in association football. J R Stat Soc Ser Gen. (1968) 131(4):581–5. 10.2307/2343726

[2] FIFA. FIFA Big Count 2006: 270 Million People Active in Football. Zürich: FIFA Communications Division, Information Services (2007). Available online at: https://digitalhub.fifa.com/m/55621f9fdc8ea7b4/original/mzid0qmguixkcmruvema-pdf.pdf (Accessed May 12, 2023).

[3] Wunderlich F. Using the wisdom of crowds in sports: how performance analysis in football can benefit from the information enclosed in betting odds. Int J Perform Anal Sport. (2024) 25(4):1–20. 10.1080/24748668.2024.2439034

[4] Hewitt J Karakuş O. A machine learning approach for player and position adjusted expected goals in football (soccer). Frankl Open. (2023) 4:4. 10.1016/j.fraope.2023.100034

[5] Mead J O’Hare A McMenemy P. Expected goals in football: improving model performance and demonstrating value. PLoS One. (2023) 18(4):1–29. 10.1371/journal.pone.0282295 PMC10075453 37018167

[6] Rathke A. An examination of expected goals and shot efficiency in soccer. J Hum Sport Exerc. (2017) 12(2):514–29. 10.14198/jhse.2017.12.Proc2.05

[7] Forcher L Altmann S Forcher L Jekauc D Kempe M. The use of player tracking data to analyze defensive play in professional soccer - A scoping review. Int J Sports Sci Coach. (2022) 17(6):1567–92. 10.1177/17479541221075734

[8] Forcher L Forcher L Altmann S Woll A. Soccer strategy tradeoff – the high-stakes risk & reward of Bundesliga transitions (based on expected ball gain & expected possession value). Int J Sport Sci Coach. (2025):0–22. (in press). 10.1177/17479541251389831

[9] Lee M Hong JG Bauer M Ko P K S. exPress: contextual valuation of individual players within pressing situations in soccer. In: MIT Sloan Sports Analytics Conference; 2025; Boston, Unites States of America. (2025). p. 1–16. Available online at: https://cdn.prod.website-files.com/5f1af76ed86d6771ad48324b/67c11b74f20ffc6e0d19bd7b_exPress_MITSloan_Final.pdf

[10] Franks A D’Amour A Cervone D Bornn L. Meta-analytics: tools for understanding the statistical properties of sports metrics. arXiv. (2016):8165–204. 10.48550/arXiv.1609.09830

[11] Dijkhuis TB Kempe M Lemmink KAPM. Early prediction of physical performance in elite soccer matches—a machine learning approach to support substitutions. Entropy. (2021) 23(8):1–15. 10.3390/e23080952 PMC8394038 34441092

[12] Berrar D Lopes P Dubitzky W. A data- and knowledge-driven framework for developing machine learning models to predict soccer match outcomes. Mach Learn. (2024) 113(10):8165–204. 10.1007/s10994-024-06625-9

[13] Hubáček O Šourek G Železnỳ F. Forty years of score-based soccer match outcome prediction: an experimental review. IMA J Manag Math. (2022) 33(1):1–18. 10.1093/imaman/dpab029

[14] Wunderlich F Memmert D. Forecasting the outcomes of sports events: a review. Eur J Sport Sci. (2021) 21(7):944–57. 10.1080/17461391.2020.1793002 32628066

[15] Dixon MJ Coles SG. Modelling association football scores and inefficiencies in the football betting market. J R Stat Soc Ser C Appl Stat. (1997) 46(2):265–80. 10.1111/1467-9876.00065

[16] Klemp M Wunderlich F Memmert D. In-play forecasting in football using event and positional data. Sci Rep. (2021) 11(1):24139. 10.1038/s41598-021-03157-3 34921155 PMC8683419

[17] O’Donoghue PG Dubitzky W Lopes P Berrar D Lagan K Hassan D An evaluation of quantitative and qualitative methods of predicting the 2002 FIFA world cup. J Sports Sci. (2004) 22(6):513–4. 10.1080/02640410410001675423

[18] Berrar D Lopes P Dubitzky W. Incorporating domain knowledge in machine learning for soccer outcome prediction. Mach Learn. (2019) 108(1):97–126. 10.1007/s10994-018-5747-8

[19] Angelini G De Angelis L. PARX model for football match predictions. J Forecast. (2017) 36(7):795–807. 10.1002/for.2471

[20] Lepschy H Waesche H Woll A. Success factors in football: an analysis of the German Bundesliga. Int J Perform Anal Sport. (2020) 20(2):150–64. 10.1080/24748668.2020.1726157

[21] Rue H Salvesen Y. Prediction and retrospective analysis of soccer matches in a league. J R Stat Soc Ser - Stat. (2000) 49(3):399–418.

[22] DFL. Definitionskatalog Offizielle Spieldaten (Definitions for Official Gama Data). Frankfurt: DFL: Definitionskatalog Offizielle Spieldaten (2014).

[23] Linke D Link D Lames M. Football-specific validity of TRACAB’s optical video tracking systems. PLoS One. (2020) 15:1–17. 10.1371/journal.pone.0230179 PMC7064167 32155220

[24] Anzer G Bauer P. A goal scoring probability model for shots based on synchronized positional and event data in football (soccer). Front Sports Act Living. (2021) 3:1–15. 10.3389/fspor.2021.624475 PMC8056301 33889843

[25] Cavus M Biecek P. Explainable expected goal models for performance analysis in football analytics. 2022 IEEE 9th International Conference on Data Science and Advanced Analytics (DSAA). IEEE (2022). p. 1–9. Available online at: https://ieeexplore.ieee.org/abstract/document/10032440/ (Accessed April 2, 2024).

[26] Fernández J Bornn L Cervone D. A framework for the fine-grained evaluation of the instantaneous expected value of soccer possessions. Mach Learn. (2021) 110(6):1389–427. 10.1007/s10994-021-05989-6 34759466 PMC8570314

[27] Bauer P. Automated detection of Complex tactical patterns in football—using machine learning techniques to identify tactical behavior (Phd thesis). Eberhard Karls Universität Tübingen, Tübingen, Germany (2022). Available online at: https://publikationen.uni-tuebingen.de/xmlui/handle/10900/124679

[28] Forcher L. Success factors in soccer defense – match analysis in soccer based on positional tracking data (Phd thesis). Karlsruher Institute of Technology (KIT), Karlsruhe, Germany (2024). Available online at: https://publikationen.bibliothek.kit.edu/1000173445 (Accessed August 27, 2024).

[29] Forcher L Beckmann T Wohak O Romeike C Graf F Altmann S. Prediction of defensive success in elite soccer using machine learning - tactical analysis of defensive play using tracking data and explainable AI. Sci Med Footb. (2024) 8(4):317–32. 10.1080/24733938.2023.2239766 37477376

[30] Marcílio WE Eler DM. From explanations to feature selection: assessing SHAP values as feature selection mechanism. In: 33rd Conference on Graphics, Patterns and Images (SIBGRAPI). IEEE (2020). p. 340–7

[31] Maher MJ. Modelling association football scores. Stat Neerl. (1982) 36(3):109–18. 10.1111/j.1467-9574.1982.tb00782.x

[32] Karlis D Ntzoufras I. Analysis of sports data by using bivariate poisson models. J R Stat Soc Ser Stat. (2003) 52(3):381–93. 10.1111/1467-9884.00366

[33] Ley C Wiele TVD Eetvelde HV. Ranking soccer teams on the basis of their current strength: a comparison of maximum likelihood approaches. Stat Model. (2019) 19(1):55–73. 10.1177/1471082X18817650

[34] Wunderlich F Memmert D. The betting odds rating system: using soccer forecasts to forecast soccer. PLoS One. (2018) 13(6):e0198668. 10.1371/journal.pone.0198668 29870554 PMC5988281

[35] Davidson RR. On extending the bradley-terry model to accommodate ties in paired comparison experiments. J Am Stat Assoc. (1970) 65(329):317–28. 10.1080/01621459.1970.10481082

[36] Szczecinski L Djebbi A. Understanding draws in elo rating algorithm. J Quant Anal Sports. (2020) 16(3):211–20. 10.1515/jqas-2019-0102

[37] Murphy AH. The ranked probability score and the probability score: a comparison. Mon Weather Rev. (1970) 98(12):917–24. 10.1175/1520-0493(1970)098<0917:TRPSAT>2.3.CO;2

[38] Stafylidis A Chatzinikolaou K Mandroukas A Stafylidis C Michailidis Y Metaxas TI. First to score, first to win? Comparing match outcomes and developing a predictive model of success using performance metrics at the FIFA club world cup 2025. Appl Sci. (2025) 15(15):8471. 10.3390/app15158471

[39] Stafylidis A Mandroukas A Michailidis Y Metaxas TI. Decoding success: predictive analysis of UEFA euro 2024 to uncover key factors influencing soccer match outcomes. Appl Sci. (2024) 14(17):7740. 10.3390/app14177740

[40] Pollard R. Home advantage in soccer: variations in its magnitude and a literature review of the inter-related factors associated with its existence. J Sport Behav. (2006) 29(2):169.

[41] France JJ. Examination of goals scored in the 2022 world cup football tournament in Qatar. J Phys Educ Sport. (2023) 23(11):2951–62. 10.7752/jpes.2023.11336

[42] Mićović B Leontijević B Dopsaj M Janković A Milanović Z Garcia Ramos A. The Qatar 2022 world cup warm-up: football goal-scoring evolution in the last 14 FIFA world cups (1966–2018). Front Psychol. (2023) 13:1–10. 10.3389/fpsyg.2022.954876 PMC9846231 36687951

[43] Cavus M Stando A Biecek P. Glocal explanations of expected goal models in soccer. arXiv. (2023):1–26. 10.48550/arXiv.2308.15559

[44] Cortez A Trigo A Loureiro N. Football match line-up prediction based on physiological variables: a machine learning approach. Computers. (2022) 11(3):40. 10.3390/computers11030040

